# Comparative utility of frailty to a general prognostic score in identifying patients at risk for poor outcomes after aortic valve replacement

**DOI:** 10.1186/s12877-020-1440-4

**Published:** 2020-02-03

**Authors:** Sandra Shi, Natalia Festa, Jonathan Afilalo, Jeffrey J. Popma, Kamal R. Khabbaz, Roger J. Laham, Kimberly Guibone, Dae Hyun Kim

**Affiliations:** 1 Division of Gerontology, Department of Medicine, Beth Israel Deaconess Medical Center, Harvard Medical School, Boston, MA USA; 2 Hinda and Arthur Marcus Institute for Aging Research, Hebrew Senior Life, Harvard Medical School, Boston, MA USA; 3 Department of Internal Medicine, Massachusetts General Hospital, Harvard Medical School, Boston, MA USA; 4 Centre for Clinical Epidemiology, Division of Cardiology Jewish General Hospital, McGill University, Montreal, Quebec Canada; 5 Division of Cardiology, Department of Medicine, Beth Israel Deaconess Medical Center, Harvard Medical School, Boston, MA USA; 6 Division of Cardiac Surgery, Department of Surgery, Beth Israel Deaconess Medical Center, Harvard Medical School, Boston, MA USA; 7 Division of Pharmacoepidemiology and Pharmacoeconomics, Department of Medicine, Brigham and Women’s Hospital, Harvard Medical School, Boston, MA USA

**Keywords:** Aortic valve replacement, Frailty, Mortality, Geriatric assessment

## Abstract

**Background:**

Current guidelines recommend considering life expectancy before aortic valve replacement (AVR). We compared the performance of a general mortality index, the Lee index, to a frailty index.

**Methods:**

We conducted a prospective cohort study of 246 older adults undergoing surgical (SAVR) or transcatheter aortic valve replacement (TAVR) at a single academic medical center. We compared performance of the Lee index to a deficit accumulation frailty index (FI). Logistic regression was used to assess the association of Lee index or FI with poor outcome, defined as death or functional decline with severe symptoms at 12 months. Discrimination was assessed using C-statistics.

**Results:**

In the overall cohort, 44 experienced poor outcome (31 deaths, 13 functional decline with severe symptoms). The risk of poor outcome by Lee index quartiles was 6.8% (reference), 17.9% (odds ratio [OR], 3.0; 95% confidence interval, [0.9–10.2]), 20.0% (OR 3.4; [1.0–11.4]), and 34.0% (OR 7.1; [2.2–22.6]) (p-for-trend = 0.001). Risk of poor outcome by FI quartiles was 3.6% (reference), 10.3% (OR 3.1; [0.6–15.8]), 25.0% (OR 8.8; [1.9–41.0]), and 37.3% (OR 15.8; [3.5–71.1]) (p-for-trend< 0.001). The Lee index predicted the risk of poor outcome in the SAVR cohort Lee index (quartiles 1–4: 2.1, 4.0, 15.4, and 20.0%; p-for-trend = 0.04), but not in the TAVR cohort (quartiles 1–4: 27.3, 29.0, 21.3, 35.4%; p-for-trend = 0.42). In contrast, the FI did not predict the risk of poor outcome well in the SAVR cohort (quartiles 1–4: 2.3, 4.4, 15.8, and 0%; p-for-trend = 0.24), however in the TAVR cohort (quartiles 1–4: 9.1, 14.3, 29.7, and 40.7%; p-for-trend = 0.004). Compared to the Lee index, an FI demonstrated higher C-statistics in the overall (Lee index versus FI: 0.680 versus 0.735; *p* = 0.03) and TAVR (0.560 versus 0.644; *p* = 0.03) cohorts, but not SAVR cohort (0.724 versus 0.766; *p* = 0.09).

**Conclusions:**

While a general mortality index Lee index predicted death or functional decline with severe symptoms at 12 months well among SAVR patients, the FI derived from a multi-domain geriatric assessment better informs risk-stratification for high-risk TAVR patients.

## Introduction

Aortic stenosis is a disease disproportionately affecting older adults, expected to increase in incidence with the aging population [1]. Historically, the standard of care for this population has been surgical aortic valve replacement (SAVR), however, contemporary transcatheter aortic valve replacement (TAVR) is now an option for patients with severe aortic stenosis, who have historically not been surgical candidates and thus had no interventional options. More recently, the approval of TAVR for low-risk patients has augmented procedural volumes among healthier patients [2, 3]. Despite a dynamic risk-profile of the average TAVR candidate, there remain considerable challenges in determining procedural candidacy among the complex and multimorbid patients to whom this intervention was first offered [1]. The anticipated increase in procedural volumes prompt novel considerations in defining procedural candidacy and person-centered outcomes for high-risk individuals.

The American College of Cardiology (ACC) guidelines emphasize primary care provider roles to recognize, investigate, and appropriately refer for management of valvular heart disease [4]. In doing so, consideration of life-expectancy is recommended as part of evaluation for TAVR, to help determine futility [4]. Prognostic indices for mortality prediction have been developed and applied in the general older adult population [5, 6]. However, the developmental cohorts differ from the population of TAVR candidates with respect to age, comorbidities, and functional status. For example, the Lee index, a well validated and widely adopted 4–10 year prognostic index for mortality was validated among community-dwelling individuals with a median age of less than 70-years [6, 7]. Additionally prognostic indices incorporate demographic factors such as age and sex, and these are typically heavily weighted, which may limit discriminative ability in the oldest old populations. Lastly, prognostic indices to estimate mortality generally do not account for frailty, a state of diminished physiologic reserve, known to confer heightened vulnerability to adverse events in the setting of cardiac surgery [8–10]. In fact, current literature for TAVR evaluation supports risk stratification by integrating markers of frailty including gait speed and chair stands [10, 11], or comprehensive geriatric assessment [11]. Nonetheless, adoption of frailty measurements remains low in this setting; the ACC-TAVR risk score does not consider any of the frailty markers [4].

Finally, current cardiac risk stratification estimates 30-day mortality and major adverse cardiac events. However frail and multimorbid patients often value functional independence more than longevity [12]. Specifically, work in patients with heart failure had suggests a preference for preservation of quality of life [13], and TAVR patients have described preserving independence as a primary driving factor in their decisions [14, 15]. An evolution towards predicting functional outcomes may facilitate better informed decisions among older and higher-risk candidates for AVR [10, 11, 15]. Thus, how to best estimate prognosis in this population to inform treatment decisions remains uncertain. In this paper we evaluated the utility of a general prognostic instrument, the Lee index, in prediction of functional decline or death following AVR [6]. We further compare its performance characteristics to a comprehensive geriatric assessment-based frailty index (FI).

## Methods

### Study population

We conducted a prospective cohort study of older adults undergoing AVR at the Beth Israel Deaconess Medical Center, Boston, MA, USA. Study design and protocols have been previously published [9]. We prospectively enrolled a cohort of patients, aged 70-years or older, undergoing SAVR or TAVR for severe AS at a single academic medical center. Patients were excluded for 1) emergent surgery or surgery involving the aorta or another heart valve; 2) clinical instability (such as hemodynamic instability, acute decompensated heart failure, or active myocardial ischemia); 3) Mini-Mental State Examination (MMSE) score < 15 points or active psychosis; or 4) non-English speaking. In total, between 2014 and 2016, we screened 446 patients and enrolled 246. This analysis included 91 SAVR and 137 TAVR patients with available functional status data at 12 months. None of the research data collected impacted ultimate procedural decisions. This study was approved by the Institutional Review Board and written consent was obtained.

### Study measurements

A trained research assistant or research nurse interviewed patients to obtain New York Heart Association (NYHA) classification, activities of daily living (ADLs), instrumental activities of daily living (IADLs), 5 tasks in the Nagi scale, and 3 tasks in the Rosow-Breslau scale (Additional file 1: Table S1). We also measured MMSE, 5-item Geriatric Depression Scale, gait speed (m/sec) (calculated from 3 trials of 5-m walk at usual pace), and average grip strength (kg) (3 measurements using a Jamar hydraulic dynamometer in the dominant hand). A study-affiliated geriatrician reviewed medical records to extract body mass index, comorbidities, medications, and laboratory values. The Society of Thoracic Surgeons Predicted Risk of Mortality (STS-PROM) and Charlson comorbidity index were calculated.

We calculated a Lee index and FI score for each participants at the time of pre-operative assessment. The Lee index (range 0–26) is based on 12 items: age, sex, body mass index (BMI) < 25 kg/m^2^, lung disease, cancer, diabetes, congestive heart failure, current smoking, difficulty bathing, difficulty with finances, difficulty pushing or pulling large objects, and difficulty walking several blocks [6] The presence of an item assigns a given number of points, (up to 7 for age, 1 or 2 points for others). Higher points indicate a higher risk of mortality and thus worse prognosis. The FI (range 0–1) was based on the deficit accumulation model of frailty. It was calculated by the proportion of deficits among 48 items spanning 5 domains: medical comorbidities, functional limitations (ADL and IADLs), physical performance measures (gait speed, grip strength, chair stands), cognition, and nutrition (Additional file 1: Table S1) [16]. For example, if 12 deficits were present in a given individual, this individual would be assigned a FI score of 0.25 (=12/48). Greater scores indicate more advanced frailty [17].

### Outcomes

Trained research assistants conducted follow-up telephone interviews. Information was obtained via mail-in questionnaire if we were unable to reach participants by phone. We ascertained vital status, NYHA class, and limitations in 22 daily activities and physical tasks. Poor outcome, our combined endpoint of interest, was defined as death, or NYHA Class III or IV (indicating symptoms at minimal activity) with functional decline at 12 months.

### Statistical analysis

As TAVR patients were clinically different from SAVR patients, the cohorts were analyzed separately. However as which procedure a patient will ultimately undergo is not clear during pre-operative testing, the overall cohort was examined together as well, to provide information that may be useful for preliminary evaluation. Baseline preoperative characteristics were compared between the SAVR and TAVR cohorts using t-test or chi-square test. We created risk quartiles of the Lee index and FI based on score distributions in the combined cohort. We then calculated the percentage of patients within each risk quartile who experienced the poor outcome at 12 months and compared the proportions using a trend test. Logistic regression was used to estimate the odds ratio (OR) and 95% confidence interval (CI) of poor outcome at 12 months for both Lee index and FI quartiles in each cohort, with and without adjustment for age and sex. As a sensitivity analysis we also performed logistic regression for continuous Lee index and FI scores after standardization. We assessed discrimination for each index as a continuous variable in the combined cohort as well as SAVR and TAVR cohorts with C-statistics, compared to each other. Differences in C-statistics between models were compared with 1000 bootstrap resampling. Analyses were performed in Stata release 14 (StataCorp, College Station, TX). A 2-sided *p*-value < 0.05 was considered statistically significant.

## Results

### Cohort characteristics

Of 103 SAVR and 143 TAVR candidates who completed baseline measurements, a total of 44 had the poor outcome (5 SAVR, 39 TAVR), including 31 deaths (3 SAVR, 28 TAVR). A total of 12 SAVR and 6 TAVR participants were lost to follow up. The mean age of TAVR patients was 6.4-years older than SAVR patients (84.4-years versus 78.0-years; *p* < 0.001, Table 1). TAVR candidates had a higher mean Charlson comorbidity index score (3.6 versus 2.1; *p* < 0.001), and a greater STS-PROM (5.9% versus 2.8%; *p* < 0.001). TAVR patients had greater proportion of ADL impairment (17.0% versus 5.6%; *p* < 0.001) and IADL disability (80.0% versus 48.3%; *p* < 0.001). TAVR patients also had lower mean gait speed (0.57 versus 0.94 m/s; *p* < 0.001), and lower mean MMSE scores (25.1 versus 27.0 points; *p* < 0.001). The mean Lee index score was 9.2 in SAVR patients (range: 3–17) and 13.4 in TAVR patients (range: 7–23) (Additional file 1: Figure S1).

**Table 1:** Baseline characteristics

Characteristics	SAVR (*N* = 91)	TAVR (*N* = 137)	*P* value
Age, years, mean ± SD	78.0 ± 5.3	84.4 ± 5.8	< 0.001
Male, n (%)	50 (56%)	66 (49.0%)	0.29
Non-white race, n (%)	4 (4.5%)	2 (1.5%)	0.17
Body Mass Index, mean ± SD	29.0 ± 5.0	27.0 ± 6.4	0.02
Living at home, n (%)	87 (97.8%)	130 (96.3%)	0.54
STS predicted risk of mortality, %, mean ± SD	2.8 ± 1.4	5.9 ± 3.0	< 0.001
Heart Failure, n (%)	30 (26.8)	82 (73.2)	< 0.001
Charlson Comorbidity Index, mean ± SD	2.1 ± 1.7	3.6 ± 2.3	< 0.001
MMSE score, points, mean ± SD	27.0 ± 2.5	25.1 ± 3.2	< 0.001
Gait speed, m/sec, mean ± SD	0.94 ± 0.35	0.57 ± 0.22	< 0.001
ADL disability, n (%)	5 (5.6%)	23 (17.0%)	0.01
IADL disability, n (%)	43 (48.3%)	108 (80.0%)	< 0.001
NYHA Class 3 or 4, n (%)	58 (32.6)	120 (67.4)	< 0.001
Lee index Score, mean ± SD	9.2 ± 3.1	13.4 ± 3.2	< 0.001
Frailty Index (FI), mean ± SD	0.24 ± 0.11	0.38 ± 0.11	< 0.001

Note: *Abbreviations*: *ADL* activities of daily living, *IADL* instrumental activities of daily living, *MMSE* mini mental state exam, *NYHA* New York Heart Association, *SAVR* surgical aortic valve replacement, *SD* standard deviation, *STS* Society of Thoracic Surgeons, *TAVR* transcatheter aortic valve replacement

### Risk of poor outcomes according to the Lee index categories

The risk of poor outcome in the combined cohort was 6.8% in quartile 1 (reference), 17.9% in quartile 2 (OR, 3.0; 95% CI, 0.9–10.2), 20.0% in quartile 3 (OR, 3.4; 95% CI, 1.0–11.4), and 34.0% in quartile 4 (OR, 7.1; 95% CI, 2.2–22.6) (p-for-trend = 0.004) (Table 2). This positive trend between the Lee index and poor outcome remained statistically significant after adjusting for age and sex (OR 2.7 [95% CI, 0.8–9.5] in quartile 2, OR 2.8 [95% CI, 0.8–10.5] in quartile 3, and OR 6.0 [95% CI, 1.5–23.3] in quartile 4, p-for-trend = 0.01).

**Table 2:** Risk of poor outcome at 12 months by Lee index quartiles

Population	Quartile 1 (0–9 points)	Quartile 2 (10–11 points)	Quartile 3 (12–14 points)	Quartile 4 (≥15 points)	P-for-trend
Combined, n/N (%) 44/228 (19.3%)	4/59 (6.8%)	10/56 (17.9%)	12/60 (20.0%)	18/53 (34.0%)	
Unadjusted OR (95% CI)	Ref	3.0 (0.9–10.2)	3.4 (1.0–11.4)	7.1 (2.2–22.6)	0.001
Adjusted OR (95% CI)^a^	Ref	2.7 (0.8–9.5)	2.8 (0.8–10.5)	6.0 (1.5–23.3)	0.01
SAVR, n/N (%) 5/91 (5.5%)	1/48 (2.1%)	1/25 (4.0%)	2/13 (15.4%)	1/5 (20.0%)	
Unadjusted OR (95% CI)	Ref	2.0 (0.2–32.7)	8.5 (0.7–102.9)	11.8 (0.6–225.4)	0.04
Adjusted OR (95% CI)^a^	Ref	1.3 (0.1–25.2)	5.4 (0.3–116.8)	5.9 (0.1–236.6)	0.28
TAVR, n/N (%) 39/137 (28.5%)	3/11 (27.3%)	9/31 (29.0%)	10/47 (21.3%)	17/48 (35.4%)	
Unadjusted OR (95% CI)	Ref	1.1 (0.2–5.1)	0.7 (0.2–3.2)	1.4 (0.3–6.3)	0.42
Adjusted OR (95% CI)^a^	Ref	1.1 (0.2–5.2)	0.8 (0.2–3.6)	1.4 (0.3–7.2)	0.56

^a^Note: adjusted models include age and sex

*Abbreviations*: *OR* odds ratios, *SAVR* surgical aortic valve replacement, *TAVR* transcatheter aortic valve replacement

In the SAVR cohort, the risk of poor outcome was 2.1% in quartile 1 (reference), 4.0% in quartile 2 (OR, 2.0; 95% CI, 0.2–32.7), 15.4% in quartile 3 (OR, 8.5; 95% CI, 0.7–102.9), and 20.0% in quartile 4 (OR, 11.8; 95% CI, 0.6–225.4) (p-for-trend = 0.13). This trend attenuated after adjustment for age and sex (p-for-trend = 0.28).

In the TAVR cohort, the risk of poor outcome was 27.3% in quartile 1 (reference), 29.0% (OR, 1.1; 95% CI, 0.2–5.1), 31.3% (OR, 0.7; 95% CI, 0.2–3.2), and 35.4% (OR, 1.4; 95% CI, 0.3–6.3) (p-for-trend = 0.42). There was not a statistically significant trend between the Lee index and poor outcome after adjustment for age and sex (p-for-trend = 0.56). Sensitivity analyses performed standardizing the Lee index did not appreciably change results (Additional file 1: Table S2).

### Prediction of poor outcomes with FI

The risk of poor outcome in the combined cohort was 3.6% in quartile 1 (reference), 10.3% in quartile 2 (OR, 3.1; 95% CI, 0.6–15.8), 25.0% in quartile 3 (OR, 8.8; 95% CI, 1.9–41.0), and 37.3% in quartile 4 (OR, 15.8; 95% CI, 3.5–71.1) (p-for-trend< 0.001) (Table 3). This positive trend between the FI and poor outcome remained statistically significant after adjusting for age and sex (OR 2.6 [0.5–13.9], OR 7.2 [1.5–34.5], OR 13.2 [2.8–61.1] in increasing risk quartiles; p-for-trend< 0.001).

**Table 3:** Risk of poor outcome at 12 months by FI quartiles

Population	Quartile 1 (FI < 0.23)	Quartile 2 (FI 0.23–0.31)	Quartile 3 (FI 0.32–0.40)	Quartile 4 (FI ≥ 0.41)	P-for-trend
Combined, n/N (%) 44/228 (19.3%)	2/55 (3.6%)	6/58 (10.3%)	14/56 (25.0%)	22/59 (37.3%)	
Unadjusted OR (95% CI)	Ref	3.1 (0.6–15.8)	8.8 (1.9–41.0)	15.8 (3.5–71.1)	< 0.001
Adjusted OR (95% CI)^a^	Ref	2.6 (0.5–13.9)	7.2 (1.5–34.5)	13.2 (2.8–61.1)	< 0.001
SAVR, n/N (%) 5/91 (5.5%)	1/44 (2.3%)	1/23 (4.4%)	3/19 (15.8%)	0/5 (0%)	
Unadjusted OR (95% CI)	Ref	2.0 (0.1–32.8)	8.1 (0.8–83.3)	Not estimated	0.24
Adjusted OR (95% CI)^a^	Ref	1.3 (0.1–26.0)	4.4 (0.4–49.5)	Not estimated	0.53
TAVR, n/N (%) 39/137 (28.5%)	1/11 (9.1%)	5/35 (14.3%)	11/37 (29.7%)	22/54 (40.7%)	
Unadjusted OR (95% CI)	Ref	1.7 (0.2–16.0)	4.2 (0.5–37.2)	6.9 (0.8–57.6)	0.004
Adjusted OR (95% CI)^a^	Ref	1.6 (0.2–16.0)	3.9 (0.4–34.8)	6.6 (0.8–55.9)	0.004

^a^Note: adjusted models include age and sex

*Abbreviations*: *CI* confidence interval, *FI* frailty index, *OR* odds ratios, *SAVR* surgical aortic valve replacement, *TAVR* transcatheter aortic valve replacement

In the SAVR cohort, the risk of poor outcome was 2.3% in quartile 1 (reference), 4.4% in quartile 2 (OR, 2.0; 95% CI, 0.1–32.8), 15.8% in quartile 3 (OR, 8.1; 95% CI, 0.8–83.3), and 0% in quartile 4 (p-for-trend = 0.24). This trend attenuated after adjustment for age and sex (OR 1.3 [0.1–26.0], OR 4.4 [0.4–49.5] in quartile 2 and 3 respectively) (p-for-trend = 0.53).

In the TAVR cohort, the risk of poor outcome was 9.1% in quartile 1 (reference), 14.3% in quartile 2 (OR, 1.7; 95% CI, 0.2–16.0), 29.7% in quartile 3 (OR, 4.2; 95% CI, 0.5–37.2), and 40.7% in quartile 4 (OR, 6.9; 95% CI, 0.8–57.6) (p-for-trend = 0.004). This trend remained after adjustment for age and sex, with (OR 1.6 [0.2–16.0] in quartile 2, OR 3.9 [0.4–34.8] in quartile 3, and OR 6.6 [0.8–55.9] in quartile 4; p-for-trend = 0.004).

### Comparison of model discrimination

**Fig. 1:**
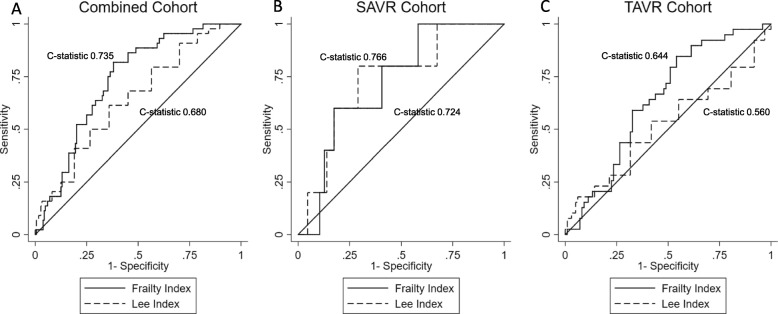
Comparison of Receiver Operator Characteristic Curves for Lee index and FI for prediction of poor outcome at 12 months. Abbreviations: SAVR – Surgical Aortic Valve Replacement. TAVR – Transcatheter Aortic Valve Replacement. In the combined cohort (panel **a**), the frailty index (FI) has a higher C-statistic than the Lee index. In SAVR cohort (panel **b**), there was no statistically significant difference between the Lee index and FI. In TAVR cohort (panel **c**), FI performed better than the Lee index

In the combined cohort the Lee index model demonstrated improved discriminatory power over the reference models (C-statistic 0.680, Fig. 1a), but not in the SAVR (C-statistic 0.766) or TAVR (C-statistic 0.560) cohorts (Fig. 1b). The FI model demonstrated improved discriminatory power within the combined (C-statistic 0.735) and TAVR (C-statistic 0.644) cohorts, but not SAVR (C-statistic 0.724).

The FI C-statistic was significantly better than the Lee index in both the combined (*p* = 0.03) and TAVR (*p* = 0.03) cohorts after adjusting for age and sex (Fig. 1). However there was not a statistically significant difference in the C-statistics between the Lee index and FI in the SAVR cohort (*p* = 0.09).

## Discussion

In this study of 228 older adults undergoing AVR, we evaluated the performance of a general mortality index in predicting death or functional decline with severe symptoms at 12 months. We observed a skewed distribution towards higher Lee index risk scores and an associated ceiling effect of the Lee index within the TAVR cohort. While the Lee index discriminated well among the healthier SAVR cohort, predictive performance was poor among TAVR patients. In contrast, the FI predicted risk of poor outcomes well in both groups, but its performance was uniquely better among TAVR patients. Thus, by integrating multi-domain geriatric assessment, the FI better informs risk-stratification for TAVR candidates.

Although the Lee index has been a favored prognostic index across many clinical and investigational contexts, it may not be an optimal tool to assess risk in an evolving population of complex, multimorbid, and often frail, procedural candidates. The indication of a ceiling effect of the Lee index in TAVR patients may be due to the unique characteristics of patients with severe aortic stenosis. For example, the mean age of patients within our TAVR cohort (84.4-years) is 34-years older than the average person in the Health and Retirement Study (HRS) cohort used by Lee et al. (67-years) [6]. As compared to 3% of individuals in the HRS cohort, 73.2% within our TAVR population carried a heart failure diagnosis [6]. In addition, a considerable subset of our TAVR population (80%) had at least one IADL limitation, as compared to 12–16% of the HRS cohort [6]. The demonstrated ceiling effect of the Lee index within our cohort supports the exigency of prognostic indices that discriminate within multimorbid and or frail populations.

The poor performance within the TAVR cohort additionally suggests a need for prognostic models that are also capable of finer discrimination when applied to older populations with a narrower age distribution. Lee et al. reported that age explained the majority of variability in mortality, as predicted by their model [6]. Thus, the development of the Lee index within an exclusively community-dwelling population may limit its generalizability to long-term care residents and community-dwellers at risk of new institutionalization.

In addition to its poor accuracy and external validity within older and higher-risk TAVR patients, the Lee index was not optimized to predict person-centered outcomes, such as functional status. Prediction of person-centered outcomes may be especially relevant to high-risk TAVR candidates, whose decisions must weigh sizable disease-mediated mortality with previously accumulated health and functional deficits [17, 18]. In a single center analysis of patient-defined goals among TAVR candidates, only 7% of patients cited survival as their primary desired endpoint [14]. This is as compared to a majority of patients describing a desire to perform a particular activity (48%) or maintain independence (30%) [14]. As such, prognostic indices developed from the general population may also be limited in their capacity to characterize the defined priorities of higher-risk procedural candidates. Dedicated research regarding post-TAVR cognitive and functional outcomes, as well as increased representation of the oldest-old within longitudinal population health surveys, may inform more accurate and patient-centered prognostic indices for TAVR candidates.

There are limitations to this study. First, our study was conducted at a large academic medical center across a predominately Caucasian population. Therefore, the generalizability of our findings to medical centers with lower procedural volumes or distinct patient demographics merits further consideration. Second, modest sample size limits our ability to detect a potentially clinically meaningful difference in discrimination for procedure-specific cohorts. Third, our combined endpoint of death or NYHA class III or IV functional status was informed by the self-report. Nonetheless, self-reported functional status has been well-validated against objective endpoints [19]. Lastly, our analysis is predicated upon a composite outcome of symptomatic functional decline and mortality, as compared to the isolated outcome of mortality in the development of the Lee index. The use of a composite endpoint, however, captures functional outcomes, which remain often of paramount importance to older adults.

## Conclusions

The peri-procedural morbidity and mortality of TAVR have declined in accordance with the recent adoption of TAVR within healthier populations, in addition to improved procedural techniques and device technology [3, 20]. However, a sizable cohort of complex and vulnerable older adults will continue to require informed counseling as to their procedural risks and anticipated outcomes. Our analysis demonstrates prognostic indices developed from the general, community-dwelling population do not appropriately discriminate risk of poor outcomes among older and multimorbid procedural candidates with frailty. Explicit incorporation of frailty may better discriminate procedural risk high-risk populations, as compared to general prognostic instruments, Lee index and provide useful information for shared decision making.

## Supplementary information

**Additional file 1: Table S1.** Comprehensive Geriatric Assessment-Based Frailty Index. **Figure S1.** Distribution of Risk Index scores in Overall Cohort and by Procedural Cohort. **Table S2.** Association between standardized index scores and poor outcome at 12 months.

## Data Availability

The datasets used and/or analyzed during the current study are available from the corresponding author on reasonable request.

## Abbreviations

ACC: American College of Cardiology
ADL: Activities of Daily Living
AVR: Aortic Valve Replacement
BMI: Body Mass Index
FI: Frailty Index
IADL: Instrumental Activities of Daily Living
MMSE: Mini-Mental State Examination
NYHA: New York Heart Association
SAVR: Surgical Aortic Valve Replacement
TAVR: Transcatheter Aortic Valve Replacement

## References

[1] Carroll JD (2016). TAVR prognosis, aging, and the second TAVR tsunami: insights from France. J Am Coll Cardiol.

[2] Mack MJ, Leon MB, Thourani VH, Makkar R, Kodali SK, Russo M (2019). Transcatheter Aortic-Valve Replacement with a Balloon-Expandable Valve in Low-Risk Patients. N Engl J Med.

[3] Gaede L, Blumenstein J, Liebetrau C, Dörr O, Kim W-K, Nef H (2018). Outcome after transvascular transcatheter aortic valve implantation in 2016. Eur Heart J.

[4] Nishimura Rick A., O’Gara Patrick T., Bavaria Joseph E., Brindis Ralph G., Carroll John D., Kavinsky Clifford J., Lindman Brian R., Linderbaum Jane A., Little Stephen H., Mack Michael J., Mauri Laura, Miranda William R., Shahian David M., Sundt Thoralf M. (2019). 2019 AATS/ACC/ASE/SCAI/STS Expert Consensus Systems of Care Document: A Proposal to Optimize Care for Patients With Valvular Heart Disease. Journal of the American College of Cardiology.

[5] Yourman LC, Lee SJ, Schonberg MA, Widera EW, Smith AK (2012). Prognostic indices for older adults: a systematic review. JAMA..

[6] Lee SJ, Lindquist K, Segal MR, Covinsky KE (2006). Development and validation of a prognostic index for 4-year mortality in older adults. JAMA..

[7] Mohile Supriya G., Dale William, Somerfield Mark R., Schonberg Mara A., Boyd Cynthia M., Burhenn Peggy S., Canin Beverly, Cohen Harvey Jay, Holmes Holly M., Hopkins Judith O., Janelsins Michelle C., Khorana Alok A., Klepin Heidi D., Lichtman Stuart M., Mustian Karen M., Tew William P., Hurria Arti (2018). Practical Assessment and Management of Vulnerabilities in Older Patients Receiving Chemotherapy: ASCO Guideline for Geriatric Oncology. Journal of Clinical Oncology.

[8] Kim Dae Hyun, Kim Caroline A., Placide Sebastian, Lipsitz Lewis A., Marcantonio Edward R. (2016). Preoperative Frailty Assessment and Outcomes at 6 Months or Later in Older Adults Undergoing Cardiac Surgical Procedures. Annals of Internal Medicine.

[9] Afilalo J, Lauck S, Kim DH, Lefèvre T, Piazza N, Lachapelle K, et al. Frailty in Older Adults Undergoing Aortic Valve Replacement: The FRAILTY-AVR Study. J Am Coll Cardiol. 2017;70:689–700. 10.1016/j.jacc.2017.06.024 28693934

[10] Shi S, Afilalo J, Lipsitz LA, Popma JJ, Khabbaz KR, Laham RJ, Guibone K, Grodstein F, Lux E, Kim DH. Frailty Phenotype and Deficit Accumulation Frailty Index in Predicting Recovery After Transcatheter and Surgical Aortic Valve Replacement. J Gerontol A Biol Sci Med Sci. 2018; PMID: 30165422. 10.1093/gerona/gly196 PMC6625585 30165422

[11] Hosler Quinn P., Maltagliati Anthony J., Shi Sandra M., Afilalo Jonathan, Popma Jeffrey J., Khabbaz Kamal R., Laham Roger J., Guibone Kimberly, Kim Dae Hyun (2019). A Practical Two‐Stage Frailty Assessment for Older Adults Undergoing Aortic Valve Replacement. Journal of the American Geriatrics Society.

[12] Fried TR, Tinetti ME, Iannone L, O’Leary JR, Towle V, Van Ness PH (2011). Health outcome prioritization as a tool for decision making among older persons with multiple chronic conditions. Arch Intern Med.

[13] Kraai IH, Vermeulen KM, Luttik MLA, Hoekstra T, Jaarsma T, Hillege HL (2013). Preferences of heart failure patients in daily clinical practice: quality of life or longevity?. Eur J Heart Fail.

[14] Coylewright M, Palmer R, O’Neill ES, Robb JF, Fried TR (2016). Patient-defined goals for the treatment of severe aortic stenosis: a qualitative analysis. Health Expect.

[15] Kim Dae Hyun, Afilalo Jonathan, Shi Sandra M., Popma Jeffrey J., Khabbaz Kamal R., Laham Roger J., Grodstein Francine, Guibone Kimberly, Lux Eliah, Lipsitz Lewis A. (2019). Evaluation of Changes in Functional Status in the Year After Aortic Valve Replacement. JAMA Internal Medicine.

[16] Searle SD, Mitnitski A, Gahbauer EA, Gill TM, Rockwood K. A standard procedure for creating a frailty index. BMC Geriatr. 2008;8:24. 10.1186/1471-2318-8-24. 10.1186/1471-2318-8-24 PMC2573877 18826625

[17] Rockwood K, Mitnitski A (2011). Frailty defined by deficit accumulation and geriatric medicine defined by frailty. Clin Geriatr Med.

[18] Leon MB, Smith CR, Mack M, Miller DC, Moses JW, Svensson LG (2010). Transcatheter aortic-valve implantation for aortic stenosis in patients who cannot undergo surgery. N Engl J Med.

[19] Young Y, Boyd CM, Guralnik JM, Fried LP (2010). Does self-reported function correspond to objective measures of functional impairment?. J Am Med Dir Assoc.

[20] Pilgrim T, Franzone A, Stortecky S, Nietlispach F, Haynes AG, Tueller D, et al. Predicting mortality after Transcatheter aortic valve replacement: external validation of the Transcatheter valve therapy registry model. Circ Cardiovasc Interv. 2017;10. 10.1161/CIRCINTERVENTIONS.117.005481. 10.1161/CIRCINTERVENTIONS.117.005481 29127116

