# Supplementary material for: Comparative utility of frailty to a general prognostic score in identifying patients at risk for poor outcomes after aortic valve replacement
Source: BMC Geriatr. 2020 Feb 3;20:38. doi: 10.1186/s12877-020-1440-4 (PMC6998298; doi:10.1186/s12877-020-1440-4)
Supplement: Supplementary file 1 — Additional file 1: Table S1. Comprehensive Geriatric Assessment-Based Frailty Index. Figure S1. Distribution of Risk Index scores in Overall Cohort and by Procedural Cohort. Table S2. Association between standardized index scores and poor outcome at 12 months. [file 12877_2020_1440_MOESM1_ESM.docx]

**Supplementary Table 1. Comprehensive Geriatric Assessment-Based Frailty Index**

| Domain | Variable | Scoring |
| --- | --- | --- |
| *Medical history* | | |
| Cardiovascular | Angina | 1 |
|  | Atrial fibrillation or flutter | 1 |
|  | Congestive heart failure | 1 |
|  | Coronary artery disease (based on cardiac catheterization) | 1 |
|  | Diabetes (history or use of insulin or oral hypoglycemic drugs) | 1 |
|  | Hypertension (history, blood pressure ≥140/90 mmHg, or use of antihypertensive drugs) | 1 |
|  | Myocardial infarction | 1 |
|  | Peripheral vascular disease | 1 |
|  | Stroke or transient ischemic attack | 1 |
| Non-cardiovascular | Anxiety | 1 |
|  | Arthritis | 1 |
|  | Asthma | 1 |
|  | Cancer diagnosed within 5 years or metastatic cancer | 1 |
|  | Chronic kidney disease (estimated GFR <60 ml/min) | 1 |
|  | Chronic obstructive pulmonary disease | 1 |
|  | Degenerative spine disease (sciatica or spinal stenosis) | 1 |
|  | Depression (medical history or 5-item GDS score ≥2) | 1 |
|  | Fall in the past year | 1 |
|  | Sensory impairment (hearing or vision impairment) | 1 |
| Medications | Use of ≥5 prescription drugs | 1 |
| *Functional limitations* | | |
| ADL items | Need personal help for feeding | 1 |
|  | Need personal help for dressing and undressing | 1 |
|  | Need personal help for grooming | 1 |
|  | Need personal help or a walker for ambulating | 1 |
|  | Need personal help for getting in and out of bed | 1 |
|  | Need personal help for bathing or shower | 1 |
|  | Need personal help for using toilet | 1 |
| IADL items | Need personal help for using telephone | 1 |
|  | Need personal help for using transportation | 1 |
|  | Need personal help for shopping | 1 |
|  | Need personal help for preparing own meals | 1 |
|  | Need personal help for housework | 1 |
|  | Need personal help for taking own medications | 1 |
|  | Need personal help for managing own money or paying bills | 1 |
| Nagi items | Difficulty pulling or pushing large objects (e.g., living room chair) | 1 |
|  | Difficulty with stooping, crouching, or kneeling | 1 |
|  | Difficulty with lifting or carrying 10 lbs (e.g., heavy bag of groceries) | 1 |
|  | Difficulty with reaching or extending arms above shoulder level | 1 |
|  | Difficulty with writing or handling small objects | 1 |
| Rosow-Breslau items | Unable to walk up or down a flight of stairs | 1 |
|  | Unable to walk half a mile | 1 |
|  | Unable to do heavy work around the house | 1 |
| *Cognitive function* | | |
| MMSE score | 27-30 points | 0 |
|  | 24-26 points | 0.3 |
|  | 21-23 points | 0.7 |
|  | <21 points | 1 |
| *Physical performance* | | |
| Gait speed | ≥1.00 m/sec | 0 |
|  | 0.80-0.99 m/sec | 0.3 |
|  | 0.60-0.79 m/sec | 0.7 |
|  | <0.60 m/sec | 1 |
| Grip strength  (dominant hand) | (men) ≥32.0 kg / (women) ≥20.0 kg | 0 |
|  | (men) 26.0-31.9 kg / (women) 16.0-19.9 kg | 0.5 |
|  | (men) <26.0 kg / (women) <16.0 kg | 1 |
| *Nutritional status* | | |
| Low albumin | Serum albumin <3.5 g/L | 1 |
| Low weight | Body mass index <21 kg/m^2^ | 1 |
| Weight loss | Unintentional weight loss >10 lbs in the past year | 1 |
| Frailty Index (number of positive items / number of items assessed): | | |

Abbreviations: ADL, activity of daily living; GDS, Geriatric Depression Scale; IADL, instrumental activity of daily living; and MMSE, Mini-Mental State Examination.


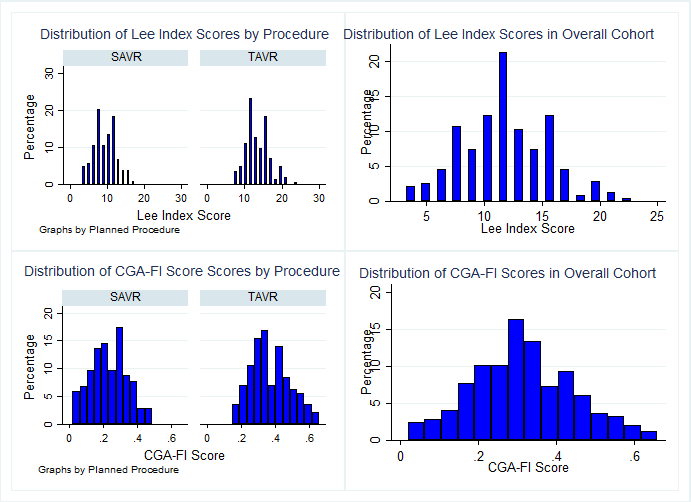
Supplemental Figure 1. Distribution of Risk Index scores in Overall Cohort and by Procedural Cohort .

**Supplemental Table 2. Association between standardized index scores and poor outcome at 12 months.**

|  | **Unadjusted Odds Ratio [95% CI]** | **Adjusted Odds Ratio [95% CI]** |
| --- | --- | --- |
| **Combined Cohort** | | |
| Standardized Lee Index | 2.03 [1.42-2.90] | 2.03 [1.31-3.14] |
| Standardized Frailty Index | 2.25 [1.55-3.26] | 2.15 [1.46-3.15] |
| **SAVR Cohort** | | |
| Standardized Lee Index | 3.05 [0.94-9.87] | 2.44 [0.50-11.85] |
| Standardized Frailty Index | 2.59 [0.77-8.70 | 2.17 [0.53-8.92] |
| **TAVR Cohort** | | |
| Standardized Lee Index | 1.38 [0.89-2.13] | 1.39 [0.84-2.30] |
| Standardized Frailty Index | 1.66 [1.07-2.57] | 1.64 [1.05-2.54] |

Note: Association of standardized scores for the Lee index and frailty index with poor outcome, defined as death or symptomatic functional decline at 12 months. Adjusted odds ratios are adjusted for age and sex. Abbreviations: SAVR, Surgical Aortic Valve Replacement; TAVR, Transcatheter Aortic Valve Replacement, CI, Confidence Interval
